# Using Celecoxib for the Suppression of Preterm Labor Instead of Magnesium Sulfate

**DOI:** 10.1155/2014/869698

**Published:** 2014-10-23

**Authors:** Najmieh Saadati, Farideh Moramezi, Maria Cheraghi, Laila Sokhray

**Affiliations:** ^1^Fertility, Infertility and Perinatology Research Center, Ahvaz Jundishapur University of Medical Sciences, Ahvaz, Iran; ^2^Social Determinant of Health Research Center, Ahvaz Jundishapur University of Medical Sciences, Ahvaz, Iran

## Abstract

We aimed to use celecoxib to suppress preterm labor instead magnesium sulfate (MgSO_4_) to prevent preterm labor.* Methods.* It was a randomized clinical trial study, which was done on 600 pregnant women. All subjects were divided into two groups by simple random sampling. One group was given 4 grams of MgSO_4_ intravenously and second group was given 100 mg of celecoxib orally every 12 hours for at least 2 days. The data were entered and analyzed using SPSS 11 and performed using *t*-test and chi-square test.* Results.* The finding of this study has shown that preterm labor may be prevented in 75.7% of subjects who had received celecoxib and there were no significant difference between two groups in frequency of history of preterm labor (*P* = 1), frequencies of nulliparity (*P* = 0.99), duration of drug use and arrest contraction (*P* = 0.29), delivery before 48 hours (*P* = 0.20), and mean gestational age in lack of response to treatment (*P* = 0.24).* Conclusions.* Result has shown that celecoxib was similar to MgSO_4_ as a medication to prevent preterm labor; it was recommended to be prescribe to prevent preterm labor, because it was cheaper than magnesium sulfate.

## 1. Introduction

Giving birth is one of the most amazing physiologic events in human life and it can be safe and pleasant in many circumstances, but in some situations it might end with known difficulties and complications for mother and her fetus. Preterm labor is one of these complications. It means regular uterine contractions with adequate frequency and intensity resulting in giving birth before 37 completed weeks of pregnancy. Preterm birth is the major reason of the neonatal death and it is responsible for about one third of neonatal mortality in the world [1, 2]. WHO publication mentions that each year more than 1 million babies die from complication of preterm delivery and the annual numbers of premature newborns are about 15 millions and this number is rising each year [3].

Based on WHO publications, UN Millennium Development Goal 4 is reduction of children mortality in two-thirds till 2015 by special attention to neonatal deaths in countries where the neonatal mortality is higher than other places. A great number of the global neonatal deaths happen in Asia [4].

Finding the ways to prevention of preterm birth is a very important issue for health care providers. There are a lot of modalities to achieve this goal but none of them has been introduced as the best way and the role of different methods in preventing preterm labor varies in different places.

Tocolytics are agents that can stop the uterine contraction. Magnesium sulphate (MgSO_4_) as the major tocolytic agent has been used by many medical centers as the first line therapy to inhibition of preterm labor. However, women receiving MgSO_4_ should be closely monitored during treatment [5]; also the severe pain and discomfort in injection site in case of IM administration is a problem with this item. On the other hand, some studies have mentioned that MgSO_4_ cannot be so effective in suppression of preterm labor [6].

Uterine contractions are produced by the effects of prostaglandins. Prostaglandins are a kind of paracrine hormones so they act where they produce. Prostaglandin producing by the decidua and fetal membranes might be one of the essential events of parturition that is followed by initiation of the uterine contraction. It seems likely that prevention of producing prostaglandins or suppression of their effects can stop preterm uterine contraction.

So in theory inhibitors of prostaglandin synthesis (including cyclooxygenase inhibitors such as celexib) are capable of arresting uterine contractility and they can prevent preterm birth [7]. Celecoxib is a sulfa nonsteroidal anti-inflammatory drug (NSAID) and selective COX-2 inhibitor. It is known under the brand name Celebrex or Celebra and Onsenal used in the treatment of inflammatory diseases and painful menstruation, menstrual symptoms, and so forth. Celecoxib is available by prescription in capsule form for oral use. Price of celexib significantly was less than MgSO_4_ price.

A study was on the comparison between MgSO_4_ and celecoxib to arrest preterm labor. Labor was arrested for 48 h in 42 (81%) and 45 (87%) of the patients in the celecoxib and magnesium sulfate groups, respectively (p-0.298) [8]. We* aimed* to use celecoxib as a cyclgenase inhibitor to suppression of preterm labor instead of magnesium sulfate (MgSO_4_) to prevent preterm labor.

## 2. Method

It was a single blinded randomized clinical trial study which had been done on 600 pregnant women with gestational age >24 weeks and <34 weeks; those had referred to Ahvaz Educational Hospitals from March to August 2013. Those patients had criteria for preterm labor diagnosis based on our Ob.-Gyn. textbook [9]. These criteria were as follows:

- at least 4th uterine contractions during 20 minutes or 8th uterine contractions during 1 hour followed by progressive changes in the cervical dilatation and effacement,
- the cervical dilatation of more than 2 cm,
- the cervical dilatation of more than 80% at the beginning.

Informed written consent was obtained from all subjects and they received preterm labor routine and maintenance therapy including bed rest and hydration (bolos infusion of 500 CC Ringer lactate during half and over, corticosteroid therapy) and if the uterine preterm contractions did not stop with these modalities, all patients were divided in two groups by simple random sampling; the first group has taken 4 gr of MgSO_4_ intravenously as loading dose and 1 gr per hour as maintenance dose till suppuration of the uterine contractions or maximum 48 hours and in this period the patients were observed for signs of MgSO_4_ toxicity by control of the urine output, deep tendon reflex, and respiratory rate. Fetal heart rate was checked regularly. Conditions of the uterine contractions were checked every 2 hours in 20-minute periods. Also the patients were observed for symptoms of headache; the patients' vital signs were checked as routine protocol. The second group had taken 100 mg celecoxib (one capsule) orally every 12 hours till suppuration of the uterine contractions or for maximum duration of 48 hours. The patients in this group were checked for nausea and vomiting and skin rush and headache.

In all subjects if the uterine preterm contractions did not stop with those medications, so immediatry drug has been stopped.

### 2.1. Inclusion Criteria for This Study

- Gestational age ≥24 weeks and ≤34 weeks with diagnosis of preterm labor based on upper criteria.

### 2.2. Exclusion Criteria for This Study

- Existing problems that interfere with normal labor process and might need early termination of pregnancy: premature preterm rapture of membrane (PPROM), evidences of placental abruption in clinical and ultrasound examination, and ultrasound, maternal fever chorioamnionitis, none reassuring fetal heart rate (FHR) patterns.
- Contraindication for stopping preterm labor uterine contraction: some maternal medical conditions such as preeclampsia, any signs of vaginal bleeding, fetus anomaly, intrauterine fetal death (IUFD), and fetal growth restriction (FGR).
- Issues related to mediations: history of being allergic to nonsteroidal anti-inflammatory drugs (NSAIDs), renal or hepatic dysfunction, concurrent use of other medications except supplements, and history of peptic ulcer.
- The patients' disagreement to enter the study.
- Stopping the uterine preterm contractions after initial preterm labor maintenance therapy.

### 2.3. Ethics

The study protocol was approved by Ethics Committee of Ahvaz Jundishapur University of Medical Sciences. All patients provided written informed consent

### 2.4. Statistical Analysis

The data were entered and analyzed using SPSS 11 (SPSS Inc., Chicago, Illinois). Age was expressed as mean (standard deviation). Other variables were expressed as frequency (%). Chi-square and independent *t*-test were used for statistical analysis. For all analyses, a significance level of *P* values < 0.05 was considered significant.

## 3. Result

The finding of this study has shown that mean age of all subjects was 26.03 ± 4.33 in MgSO_4_ group and in celecoxib group was 26.6 ± 4.01, 25.8 ± 4.63, respectively. With using independent *t*-test, there were no significant differences between mean age subjects in two groups (*P* = 0.194).

Frequencies of gestational age at time of delivery in all subjects were 31.74 ± 1.86 weeks and in MgSO_4_ group and celecoxib group were 32.1 ± 1.82 and 31.38 ± 1.83 weeks, respectively. The lowest gestational age was 27 weeks and the greatest gestational age was 34 weeks.

Frequencies of nulliparity in all pregnant women were 350 (58.3%), in MgSO_4_ group were 174 (58%), and in celecoxib group were 176 (58.7%). Frequencies of history of preterm delivery in celexib group were 12 (4%) and in MgSO_4_ group were 12 (4%).

There were no significant differences between two groups (MgSO_4_ group and celecoxib group) in frequency of history of preterm labor (*P* = 1), frequencies of nulliparity (*P* = 0.99), duration of drug use and arrest contraction (*P* = 0.29), delivery before 48 hours (*P* = 0.20), and mean gestational age in lack of response to treatment (*P* = 0.24) (Tables 1 and 2).

There was significant association between two groups (MgSO_4_ group and celecoxib group) in frequency of effacement (*P* < 0.0001), gestational age (*P* < 0.0001), and dilation (*P* = 0.06) (Table 3).

## 4. Discussion

About two-thirds of neonatal mortality are due to preterm birth, so preterm labor suppression is a big deal for health care providers. On the other hand decreasing childhood mortality is one of the WHO'S goals by 2015. Celecoxib as a cyclogenase inhibitor might be effective in preterm labor suppression.

A similar study has been done by Borna and Saeidi in 2007 and preterm labor was suppressed in 87% of MgSO_4_ group and 81% of celecoxib group so their result was similar to our result. They concluded that celecoxib is as effective as MgSO_4_ in preterm labor suppression [8, 10].

In another study by McWhorter et al. in 2004 the effects of rofecoxib and MgSO_4_ were compared with each other. They concluded that there was no difference between oral rofecoxib and intravenous magnesium sulfate in arresting preterm labor [9]. Another study compared safety of indomethacin and celecoxib to arrest preterm Labor. They reported that safety of short-term celecoxib in women with preterm labor was superior to indomethacin [10].

In this study 600 pregnant women with preterm labor criteria were randomly divided in two equal groups. MgSO_4_ was used in one group for suppression of preterm labor and the other group took celecoxib instead of MgSO_4_. 80% of MgSO_4_ group and 75% of celecoxib group showed positive response to these tocolytic agents and uterine contraction stopped at least for 48 hours and cervical dilatation and effacement did not progress in this period of time, but in this aspect; statistical calculation did not show any significant differences between two groups. In our study there were not any significant differences between cervical dilatation in two groups, but from the beginning, cervical effacement showed a significant difference between two groups; we found that although celecoxib group already had more cervical effacement, celecoxib effectiveness was as the same as MgSO_4_ group. In this study, mean time duration for stopping the uterine contraction did not show any significant differences. Cost effectiveness of celecoxib is more reasonable than MgSO_4_ and it can play a role in expenses reduction in health centers.

## 5. Study Limitations

We did not enroll the patients admitted at other hospitals especially private hospitals.

## 6. Conclusion

Celecoxib is a good alternative for MgSO_4_ as agents' tocolytic. Because it is cost less and there is better patient's compliance. Using Celecoxib no need for closes monitoring of the patients, and there are not invasive procedures such as injections and same effect of MgSO_4_.

## Figures and Tables

**Table 1:** Frequency of delivery before and after 48 hours in all subjects.

	MgSO_4_		Celecoxib		*P* value
	Frequency	%	Frequency	%	
Delivery before 48 hours	240	80	227	75.7	0.201
Delivery after 48 hours	60	20	30	24.3	

**Table 2:** Frequency of parity and history of preterm labor in two groups (MgSO_4_ group and celecoxib group).

Variables	MgSO_4_		Celecoxib		*P* value
	Frequency	%	Frequency	%	
Parity					
0	176	58.7	174	58	0.99
1	64	21.3	66	22	
2	54	18	54	18	
3	6	2	6	2	
History of preterm labor					
Yes	12	4	12	4	1
No	288	96	288	96	

**Table 3:** Frequency of gestational age, dilatation, and effacement in two groups.

Variables	MgSO_4_			Celecoxib			*P* value
	No.	Mean	SD	No.	Mean	SD	
Dilatation	300	1.83	9.26	300	1.97	8.13	0.06
Effacement	300	35.93	18.07	300	42	15.64	≤0.0001
